# Well Done! Effects of Positive Feedback on Perceived Self-Efficacy, Flow and Performance in a Mental Arithmetic Task

**DOI:** 10.3389/fpsyg.2020.01008

**Published:** 2020-06-10

**Authors:** Corinna Peifer, Pia Schönfeld, Gina Wolters, Fabienne Aust, Jürgen Margraf

**Affiliations:** ^1^Faculty of Psychology, Applied Psychology in Work, Health, and Development, Ruhr University Bochum, Bochum, Germany; ^2^Faculty of Psychology, Mental Health Research and Treatment Center, Ruhr University Bochum, Bochum, Germany

**Keywords:** feedback, self-efficacy, flow, performance, mental arithmetic

## Abstract

Self-efficacy is a well-known psychological resource, being positively associated with increased performance. Furthermore, results from field studies suggest a positive impact of self-efficacy on flow experience, which has not yet been tested experimentally. In this study, we manipulated self-efficacy by means of positive feedback and investigated whether self-efficacy serves as a mediator in the relationship between positive feedback and flow and in the relationship between positive feedback and performance. Our sample consisted of 102 participants (63 female, 39 male). The experimental group received positive feedback after completing 5 min of mental arithmetic tasks on a computer, whereas the control group received no feedback. A second session of a mental arithmetic task was then completed for 5 min. Mediation analyses confirmed that specific self-efficacy mediated a positive effect of positive feedback on flow as well as on both performance measures (quality and quantity) in a subsequent task. However, direct effects of feedback on flow and on performance were not significant, which suggests the presence of other mechanisms that remain to be investigated.

## Introduction

“Well done!” – Positive feedback has been found not only to enhance performance (e.g., Kluger and DeNisi, 1996; Hattie and Timperley, 2007), but also to be an efficient intervention to manipulate perceived self-efficacy (e.g., Brown et al., 2012). Self-efficacy refers to the judgment of one’s own abilities to successfully cope with future demands (Bandura, 1983). It can either refer to a general judgment, called general self-efficacy, or it can refer to more specific domains, such as mathematical skills, then called specific self-efficacy. Self-efficacy is well-known as a psychological resource protecting mental health and buffering the negative effects of stress (Bandura, 1977, 1986; Schönfeld et al., 2016). In line with Bandura’s social cognitive theory (SCT), there is a broad basis for higher self-efficacy being associated with lower symptoms of depression and anxiety as well as with higher optimism and emotional well-being (Benight et al., 1999; Rottmann et al., 2010; Singh and Bussey, 2011; Wang et al., 2014).

While self-efficacy has been found to enhance performance (e.g., Bandura and Locke, 2003), contradictory findings also exist, suggesting that effects may differ with respect to different performance outcomes such as performance quality and quantity (Vancouver et al., 2014). The first part of our study contributes to the answer to this yet open research question by examining the effects of positive feedback as an intervention to manipulate self-efficacy and we test how self-efficacy affects performance quality and quantity. Further, we contribute to research on the mechanisms via which the effects of positive feedback are transmitted to performance by using self-efficacy as a mediator in the feedback-performance relationship.

The second part of this study examines flow – the experience of being fully absorbed in a task (Csikszentmihalyi, 1975) – in relation to positive feedback and self-efficacy. Feedback has been described as a central antecedent of flow (e.g., Landhäußer and Keller, 2012), but effects of positive feedback on flow have not yet been investigated experimentally. Another antecedent of flow as identified in field studies (e.g., Zubair and Kamal, 2015a, b) is self-efficacy. This study aims to replicate the positive relationship between self-efficacy and flow in an experimental setting, in which self-efficacy is manipulated using positive feedback. Finally, and adding to the existing research, we aim to test whether self-efficacy transmits effects of positive feedback on flow experience.

### Positive Feedback and Performance

Feedback can be defined as the “provision of information regarding some aspect(s) of one’s task performance” (Kluger and DeNisi, 1996, p. 255). Meta-analyses show impressive effects of feedback on increased performance, with average effect sizes of *d* = 0.40 (Kluger and DeNisi, 1996) and *d* = 0.79 (Hattie and Timperley, 2007). Research has identified moderators of the feedback-performance relationship, with findings suggesting that positive feedback is more efficient than negative feedback. For example, Arbel et al. (2014) found that positive feedback improved learning performance more than negative feedback. Furthermore, it has been found that feedback after good trials enhanced learning in comparison to feedback after poor trials (Chiviacowsky and Wulf, 2007). In line with these findings, the meta-analysis of Kluger and DeNisi (1996) found that feedback following correct results, that is, positive feedback, was more effective than feedback following incorrect results. Furthermore, feedback was more effective when it was provided by a computer (*d* = 0.41) vs. not (*d* = 0.23; Kluger and DeNisi, 1996).

One particular type of feedback is normative feedback, which refers to information on one’s performance compared to referenced others, allowing comparative inferences (Hartwell and Campion, 2016). Accordingly, positive normative feedback is the information that one’s performance was better than that of referenced others, such as feedback indicating above-average performance. Studies suggest that such positive normative feedback – even if it is false feedback – leads to increased performance compared to negative normative feedback (i.e., indicating below-average performance; Bandura and Jourden, 1991; Wulf et al., 2010). Based on these findings, we expect to replicate earlier studies by finding positive effects of positive normative feedback on performance.

### Positive Feedback as an Intervention to Increase Self-Efficacy

Previous research has shown that false normative positive feedback not only affects performance, but also self-efficacy, and such feedback has been successfully applied to manipulate self-efficacy (e.g., Reynolds, 2006; Beattie et al., 2016; Brown et al., 2016; Dimotakis et al., 2017). This has also been used in experiments with mental arithmetic tasks (Weinberg et al., 1979; Wright and Gregorich, 1989; Eden and Zuk, 1995; Brown et al., 2012). The approach is in line with Bandura’s SCT (Bandura, 1977). Bandura (1977) pointed out that there are different kinds of information that lead to expectations about personal efficacy: performance accomplishment, vicarious experience, verbal persuasion and psychological states. In line with that, external persuasion through positive feedback to induce an experience of success should be an effective strategy to manipulate self-efficacy (Achterkamp et al., 2015). In line with this, we expect to replicate positive effects of positive normative feedback on self-efficacy.

### Self-Efficacy and Performance

Successful mastery experiences contribute to the development of efficacy beliefs and increase the investment of effort and the level of performance (Bandura, 1997). Perceived self-efficacy is a key dynamic and malleable factor affecting behavior (Gist and Mitchell, 1992; Hardy, 2014), and some evidence indicates that higher self-efficacy leads to better performance in cognitive and sports tasks (e.g., Beattie et al., 2014; Niemiec and Lachowicz-Tabaczek, 2015). At the same time, divergences in social cognitive and control theories lead to different assumptions about the effects of self-efficacy (see Bandura and Locke, 2003; Bandura, 2012; Schönfeld et al., 2017). For example, Powers’ (1973, 1991) perceptual control theory assumes that the discrepancy between one’s personal goal and one’s perceived progress in handling a situation successfully regulates the performed action (e.g., Vancouver et al., 2002). In case of a low discrepancy, the person will invest fewer resources in achieving the goal, and successful performance is assumed to be easy. In situations in which perceived progress is ambiguous, perceived capabilities (i.e., self-efficacy) can be used as an indicator of progress. As a consequence, high perceived skills will lead to a decreased perceived discrepancy between goal and progress. Thus, according to perceptual control theory, high self-efficacy would undermine performance and motivation. Initial empirical findings support these assumptions (e.g., Vancouver et al., 2001, 2002, 2008, 2014; Vancouver and Kendall, 2006; Vancouver, 2012; Beattie et al., 2014). A study by Vancouver et al. (2014), for example, found that self-efficacy was negatively related to performance quality, while it was positively related to performance quantity. They assumed that individuals with high self-efficacy allocate less effort per task, which leads to faster progress (performance quantity), but lower quality of results in the form of more mistakes. Yet, more research is needed to disentangle effects of self-efficacy on different performance measures.

In accordance with the findings of Vancouver et al. (2014), our study differentiates between performance quantity and performance quality in that we assume that self-efficacy has positive effects on performance quantity, but negative effects on performance quality.

A general limitation of research on self-efficacy and performance is that it is largely based on observational rather than experimental designs, so no conclusions can be drawn about the direction of effects. While most studies assume positive effects of self-efficacy on performance, Bandura (1977) in fact already identified performance accomplishment as an antecedent of self-efficacy. Accordingly, experimental research on the relationship between self-efficacy and performance is necessary to disentangle potential bidirectional effects.

Consequently, by differentiating between performance quantity and quality, and by applying an experimental design in which we manipulate self-efficacy by means of positive feedback, we aim to contribute to a better understanding of the relationship between self-efficacy and performance.

### Self-Efficacy as a Mechanism That Transmits Effects of Positive Feedback on Performance

As mentioned above, the induction of positive feedback including a favorable comparison to others has been found to be a suitable method to enhance the level of self-efficacy, which in turn, affects performance (see Zinken et al., 2008). Based on a comparative appraisal, the individual is persuaded that he or she has performed successfully, which is in line with SCT. Using feedback-manipulation as a strategy to increase a person’s appraisal of his or her capabilities, beneficial effects have also been demonstrated in the context of emotional learning processes (Zlomuzica et al., 2015). However, to the best of our knowledge, the mediation hypothesis of self-efficacy has not yet been tested. Integrating the described relationships, we propose that self-efficacy acts as a mediator, transmitting positive effects of positive feedback on performance. Taking the differential expectations for the relationship of self-efficacy with performance quantity and performance quality into account, *we expect that self-efficacy acts as a mediator, transmitting positive effects of positive feedback on performance quantity (Hypothesis 1a), but transmitting negative effects of positive feedback on performance quality (Hypothesis 1b).*

### Effects of Feedback on Flow-Experience

Flow is the positive experience of being fully absorbed in an optimally challenging task. While in flow, individuals are completely concentrated on the task at hand, which is experienced as rewarding in itself. Individuals perceive clear goals and feedback and a high level of control over the demands, thereby experiencing a merging of action and awareness, and a loss of self-consciousness, along with a distorted sense of time (Csikszentmihalyi, 1975). Flow can be experienced in different activities and tasks, among them cognitive tasks such as solving math calculations – even under laboratory conditions (e.g., Harmat et al., 2015; Ulrich et al., 2016).

Feedback has been described as one of the core antecedents fostering flow-experience (Bakker, 2005; Demerouti, 2006; Landhäußer and Keller, 2012; Nakamura and Csikszentmihalyi, 2014). This conceptualization is in line with findings based on the job characteristics model (Hackman et al., 1975), showing that feedback along with four other core job characteristics is positively related to flow experience (Bakker, 2005; Demerouti, 2006; Maeran and Cangiano, 2013). Studies that have specifically examined the relationship between feedback and flow have confirmed a positive link between the two (Rau and Riedel, 2004; Maeran and Cangiano, 2013). However, these studies have focused on feedback in general, and the effects of specifically positive feedback on flow have not yet been studied using quantitative research. Qualitative research has provided first indications that positive feedback – but not negative feedback – is an antecedent of flow (Jackson, 1995; Swann et al., 2015). In line with this, positive normative feedback has been found to have positive effects on positive affect during a challenging task (Hutchinson et al., 2008) – which is often linked to flow experience. Furthermore, positive normative feedback has been suggested to have energizing and reinforcing effects (Kühn et al., 2008) – both typical characteristics of the experience of flow. Bringing together theoretical and empirical evidence, we expect to find positive effects of positive normative feedback on flow experience.

### Self-Efficacy and Flow-Experience

A central component of flow is the perceived balance between the demands of the task and the individual’s skills (e.g., Csikszentmihalyi, 1975; Landhäußer and Keller, 2012). The level of self-efficacy is an individuals’ evaluation of his/her skills and therefore has a substantial impact on how the balance between skills and task demands is perceived. High levels of self-efficacy should thus positively impact flow. Empirical studies support this assumption: For example, Zubair and Kamal (2015a, b) investigated the relationship between the dimensions of psychological capital (Luthans et al., 2004) and flow experience and found that all dimensions, including self-efficacy, were positively related to flow. In a two-wave longitudinal study design, Salanova et al. (2006) found that work-specific self-efficacy beliefs facilitated the experience of work-related flow. In another longitudinal study, Rodríguez-Sánchez et al. (2011) found that teachers’ work-related self-efficacy positively affected their flow experience. Furthermore, collective efficacy beliefs are associated with higher flow. In a longitudinal study with small groups, it was found that collective efficacy beliefs can lead to higher collective flow, which in turn leads to higher collective self-efficacy in the future, forming a reciprocal relationship (Salanova et al., 2014). Furthermore, Pineau et al. (2014) found that self-efficacy as well as team-efficacy are significantly related to dispositional flow. All in all, abundant research supports the hypothesis that self-efficacy beliefs are positively associated with flow experience. Following the existing literature, we postulate that self-efficacy facilitates flow experience.

### Self-Efficacy as a Mechanism That Transmits Effects of Positive Feedback on Flow

While results from field studies, including long-term studies, suggest a reciprocal and positive relationship between self-efficacy and flow, this relationship has not yet been tested experimentally. As outlined above, positive feedback is an established intervention to positively affect self-efficacy. Thus, we use positive feedback to manipulate self-efficacy with the aim to test the effects of self-efficacy on flow experimentally. Furthermore, and as described above, theoretical considerations and qualitative research suggest also direct effects of positive feedback on flow. We suggest that these effects of positive feedback on flow can be explained at least partially by increased self-efficacy. Accordingly, *we propose that self-efficacy acts as a mediator, transmitting positive effects of positive feedback on flow experience (Hypothesis 2).*

## Materials and Methods

### Participants and Design

The sample was recruited at Ruhr University Bochum (Germany) through postings in social media networks, such as student groups in Facebook or via announcements on notice boards. The total sample consisted of 134 subjects (82 females, 52 males). Due to missing values, data from 23 subjects were excluded from the analyses: Eighteen participants did not complete the Flow-Short-Scale, one participant did not complete the self-efficacy scale and four participants did not perform the mental arithmetic task. The data was z-transformed and due to outliers on the study variables (flow, specific self-efficacy, performance quantity, and performance quality) another nine subjects were excluded^1^. The final analysis included data from 102 participants, of which 63 were female and 39 were male, with a mean age of 22.51 (*SD*_age_ = 3.13). Participants were mainly undergraduate students (76.5%). Another 20.6% were students with a bachelor’s degree and 2.9% held a secondary school degree. Participants rated their ability in mental arithmetic on a 100-point scale on average at *M* = 54.22 (*SD* = 17.32). Furthermore, they rated the difficulty of the experimental task on an 8-point Likert Scale from 0 = “not difficult at all” to 7 = “very difficult” to be at an average level, with the second task being slightly more difficult than the first (*M_task1_* = 3.87, *SD_task1_* = 1.60*; M_task2_* = 4.43, *SD_task2_* = 1.63).

Participants were randomly assigned to one of two conditions: the positive feedback condition (*n* = 53, 33 female, *M_age_ = 22.43, SD_age_* = 3.17) or the non-feedback condition (*n* = 49, 30 female, *M_age_* = 22.59, *SD_age_* = 3.12). All participants provided written informed consent and received course credit for participation. The study was approved by the local Ethics Committee of the Faculty of Psychology at Ruhr University Bochum, Germany.

### Task

The experimental task was a computer-based mental arithmetic task, which lasted 5 min per block, with the participant sitting alone in the laboratory in front of a computer screen. A computer program written in VB.NET (Microsoft Visual Studio [Software], 2015) was used to generate the task on the screen. Participants typed the calculated numbers into the computer and pressed “enter” after each calculation. All the previously calculated numbers could be seen on the screen while working on the task. After 5 min, the task stopped automatically. In the first block of the mental arithmetic task, participants were asked to subtract the number 12, starting at 2000, consecutively with maximal accuracy and rapidness for 5 min. In the second block of the mental arithmetic task, participants were asked to subtract the number 17, starting at 2043, consecutively with maximal accuracy and rapidness for 5 min. The mental arithmetic task was constructed based on the Trier Social Stress Test (Kirschbaum et al., 1993), which uses a similar mental arithmetic task as part of the protocol. Importantly, and in contrast to the Trier Social Stress Test, there was no social stress component in our mental arithmetic task.

### Feedback Manipulation

After the first block of the mental arithmetic task, the feedback group saw a note on their computer screen stating that their performance had been evaluated in terms of accuracy and rapidness. According to this analysis, he or she had performed better than the average of the previous participants, and that compared to the average participant, he or she was better able to follow new instructions and to manage mathematical problems spontaneously. The control group also saw a note on their screen, simply stating that time was up.

### Procedure

**FIGURE 1:**
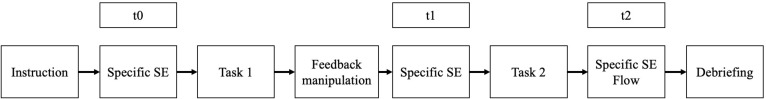
Study procedure.

The experiment was conducted in a laboratory room of Ruhr University Bochum. The participant was seated in front of a computer and asked to read and sign the informed consent form. Self-report measures and subsequent instructions were presented on the computer screen (see Figure 1). After a baseline measure of self-efficacy, the participant was asked to complete the first block of the mental arithmetic task (2000–12). Participants in the feedback group received positive normative feedback after the task was accomplished, while participants in the no-feedback group received no feedback. Right after this feedback, specific self-efficacy was assessed. Participants then completed the second block of mental arithmetic tasks (2043–17) for 5 min and finally were asked to answer questionnaires on flow and specific self-efficacy with respect to that task. Participants were then debriefed regarding the purpose of the study and the feedback manipulation.

### Measures

#### Specific Self-Efficacy

Based on a guide for constructing self-efficacy scales developed by Bandura (2006), participants rated their ability to complete mental arithmetic tasks on a 10-point scale from 0 = “cannot do at all” to 100 = “can do very well” to measure their level of specific self-efficacy (*M_t__1_* = 52.84, *SD_t__1_* = 18.21) for mental arithmetic tasks. This item was assessed at baseline level (t0), after the first task following the feedback manipulation (t1), and after the second task (compare Figure 1). The three measurement points t0, t1, and t2 were used for the manipulation check, that is, to test if the feedback manipulation increased specific self-efficacy over time. Specific self-efficacy at t1 was used to test the hypothesized mediation effects.

#### Performance

Performance quantity (*M_QN_* = 28.33, *SD_QN_* = 9.28) was assessed using the number of calculated results in the given time (5 min). Performance quality (*M_QL_* = 0.90, *SD_QL_* = 0.10) was assessed using the ratio between the number of correctly calculated results and the total number of calculated results.

#### Flow

Flow (*M_t__2_* = 4.50, *SD_t__2_* = 1.18) was measured with the Flow-Short-Scale (Rheinberg et al., 2003), which comprises ten items measuring absorption (“I did not notice time passing”) and fluency (“My thoughts/activities ran fluidly and smoothly”) as experienced during the task on a 7-point Likert Scale. The reliability of the scale was very good with a Cronbach’s Alpha of 0.92. The scale was administered after the second task (at t2; compare Figure 1).

### Data Analysis

Data were analyzed with the IBM SPSS statistics package. To analyze the efficiency of our manipulation, we tested whether participants’ specific self-efficacy increased over time in the feedback group using a repeated measures ANOVA. As the Mauchly test of sphericity was significant, we used the Greenhouse-Geisser correction procedure. The mediation analyses were conducted with the SPSS macro Process (Hayes, 2013). For the mediation analyses, all variables were z-standardized. To estimate if self-efficacy served as the mediator, the indirect effect *ab* was estimated (Preacher and Hayes, 2008). We report 95% confidence bootstrap intervals for the indirect effect (n_bootstrap_ = 5000).

## Results

Table 1 shows means, standard deviations and correlations of all study variables.

**TABLE 1:** Shows means, standard deviations and correlations of all study variables.

**Variable**	**MW**	**SD**	**MW_CG_**	**SD_CG_**	**MW_EG_**	**SD_EG_**	**1**	**2**	**3**	**4**	**5**	**6**
1 Group	–	–	–	–	–	–	1					
2 Specific SE (t0)	54.22	17.32	54.69	16.85	53.77	17.89	−0.03	1				
3 Specific SE (t1)	52.84	18.21	48.78	17.75	56.60	17.96	0.22*	0.76**	1			
4 Specific SE (t2)	50.29	19.47	49.39	19.08	51.13	19.97	0.05	0.69**	0.79*	1		
5 Flow	4.50	1.18	4.43	1.14	4.57	1.23	0.06	0.46**	0.56**	0.67**	1	
6 Performance QL	0.90	0.10	0.90	0.09	0.90	0.11	−0.00	0.29**	0.30**	0.45**	0.40**	1
7 Performance QN	28.33	9.28	28.69	9.70	28.00	8.96	−0.04	0.39**	0.37**	0.45**	0.20*	0.32**

**^∗^p < 0.05; ^∗∗^p < 0.01.**

### Manipulation Check

**FIGURE 2:**
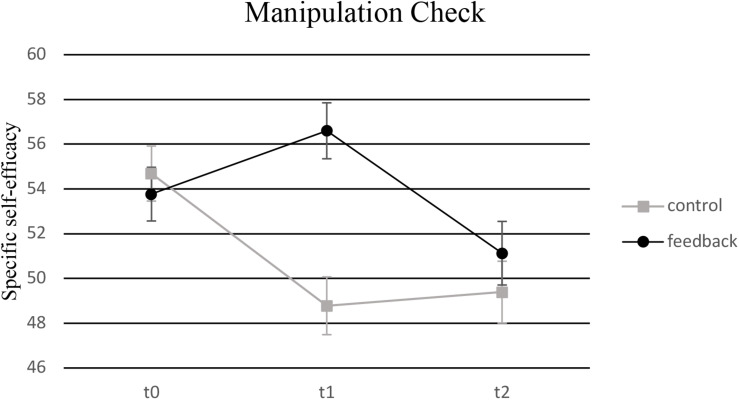
Manipulation Check: Level of specific self-efficacy regarding mental arithmetic tasks at measurement points t0, t1, and t2 for the control group (no feedback) and the experimental group (positive feedback).

With regard to the experimental manipulation of specific self-efficacy, a significant main effect of time [*F*_(__1.__82_, _181.__59__)_ = 4.97, *p* = 0.010, η*_p_*^2^ = 0.047] and an interaction effect [*F*_(__1__.82_, _18__1.59__)_ = 6.23, *p* = 0.003, η*_p_*^2^ = 0.059] were found for specific self-efficacy for mental arithmetic tasks. As can be seen in Figure 2, in contrast to the feedback group, the control group showed a decrease in specific self-efficacy for mental arithmetic tasks after Task 1.

### Testing of Hypotheses

**FIGURE 3:**
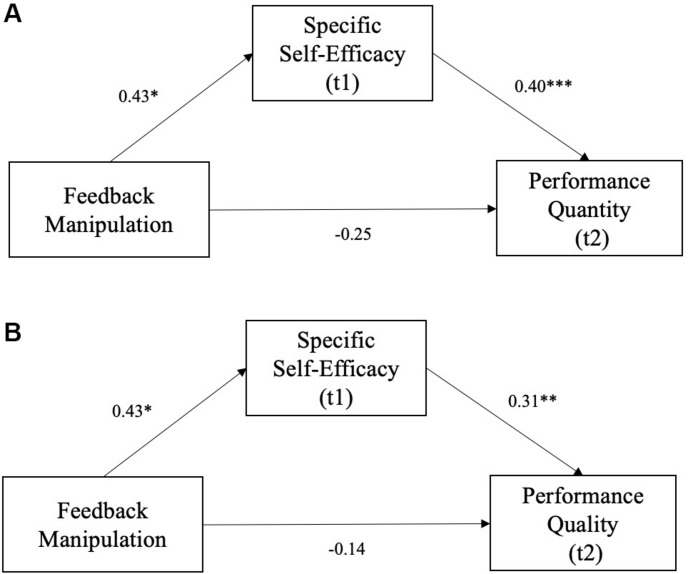
Mediation models of the effect of positive feedback on performance quantity **(A)** and performance quality **(B)** via specific self-efficacy. The arrows represent direct effects. The indirect effects were **(A)** β = 0.17 (*SE* = 0.10, 0.01 < CI < 0.39) for performance quantity and **(B)** β = 0.13 (*SE* = 0.08, 0.00 < CI < 0.32) for performance quality. *p < 0.05; **p < 0.01; ***p < 0.001.

To test *Hypothesis 1a*, we z-standardized all study variables and performed a mediation procedure with feedback as the independent variable, specific self-efficacy as the mediator, and performance quantity as the dependent variable. The *a*- (β = 0.43, *SE* = 0.19, *p* = 0.029) and *b*-path (β = 0.40, *SE* = 0.09, *p <* 0.001) were significant. The indirect effect was β = 0.17 (*SE* = 0.10, 0.01 < CI < 0.39) and significant (compare Figure 3A). The total effect was β = −0.07 (*SE* = 0.20, *t* = −0.38, *p* = 0.708) and unexpectedly *not* significant. The direct effect was also not significant (β = −0.25, *SE* = 0.19, *t* = −1.30, *p* = 0.195) when controlling for the indirect effect. However, as the indirect effect was significant, specific self-efficacy appeared to transmit a positive effect of positive feedback on performance quantity as hypothesized.

Regarding *Hypothesis 1b*, the *a*- (β = 0.43, *SE* = 0.19, *p* = 0.029) and *b*-path (β = 0.31, *SE* = 0.10, *p* = 0.002) of the model with specific self-efficacy as the mediator, group as the independent variable, and performance quality as the dependent variable were both significant. The indirect effect was β = 0.13 (*SE* = 0.08, 0.00 < CI < 0.32) and significant (compare Figure 3B). The total effect was β = −0.00 (*SE* = 0.20, *t* = −0.02, *p* = 0.988) and *not* significant. The direct effect was not significant (β = −0.14, *SE* = 0.20, *t* = −0.70, *p* = 0.483) when controlling for the indirect effect. As the b-path in this model was significantly positive, these results run counter to our expectation to find negative effects of increased self-efficacy on performance quality. We further found that self-efficacy appeared to transmit a positive effect of positive feedback on performance quality – while we had expected that a negative effect would be transmitted.

To conclude – in line with *Hypothesis 1a*, but in conflict with *Hypothesis 1b –* specific self-efficacy mediated a positive effect of positive feedback on both performance quantity and quality. Contrary to our expectations, however, the total effect of positive feedback on both performance measures was not significant.

**FIGURE 4:**
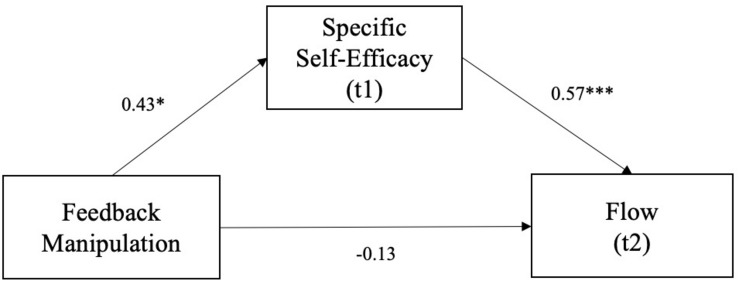
Mediation model of the effect of positive feedback on flow via specific self-efficacy. The arrows represent direct effects. The indirect effect was β = 0.25 (*SE* = 0.11, 0.02 < CI < 0.47). *p < 0.05; ***p < 0.001.

Regarding *Hypothesis 2* the *a*- (β = 0.43, *SE* = 0.19, *p* = 0.029) and *b*-path (β = 0.57, *SE* = 0.09, *p <* 0.001) of the z-standardized mediation model with specific self-efficacy as the mediator, feedback as the independent variable, and flow as the dependent variable were significant. In support of *Hypothesis 2*, the indirect effect was β = 0.25 (*SE* = 0.11, 0.02 < CI < 0.47) and significant. The total effect was β = 0.12 (*SE* = 0.20, *t* = 0.61, *p* = 0.544) and *not* significant. The direct effect was not significant (β = −0.13, *SE* = 0.17, *t* = −0.74, *p* = 0.461) when controlling for the indirect effect (compare Figure 4). The significance of the indirect effect confirms *Hypothesis 2*, that specific self-efficacy would transmit a positive effect of positive feedback on flow. However, and contrary to our expectations, the total effect of positive feedback on flow was not significant.

## Discussion

In this study we aimed to test the postulated effect of self-efficacy on flow-experience and performance experimentally. To manipulate self-efficacy, we used a well-established paradigm, that is, false normative positive feedback about performance on a mental arithmetic task. Using a bootstrap procedure to conduct mediation analyses, we found that positive feedback enhances specific self-efficacy, which, in turn, enhances performance (quality and quantity) and flow experience in a subsequent task. In the following we discuss our results in more detail:

First of all, given our successful manipulation check, we could replicate earlier studies showing that false positive normative feedback is an efficient intervention to promote self-efficacy (Weinberg et al., 1979; Wright and Gregorich, 1989; Eden and Zuk, 1995; Brown et al., 2012). It can therefore be recommended as an experimental manipulation of self-efficacy in future studies.

Furthermore, *Hypothesis 1a* was confirmed: The results suggested that positive feedback has an indirect positive effect on performance quantity via self-efficacy. Considering the positive *b*-path in our model, that is, the positive effect of self-efficacy on performance quantity, our results replicate earlier studies that also found that performance increases with increased self-efficacy (e.g., Bandura and Locke, 2003; Vancouver et al., 2014). As previous research on the relationship between self-efficacy and performance is largely based on observational data, a strength of our study is the experimental approach to manipulate self-efficacy using positive feedback.

By differentiating between performance quantity and quality, our study further contributes to the debate regarding whether – and for which performance parameters – self-efficacy has positive or maybe even negative effects on performance: some researchers argue that high self-efficacy might undermine motivation, as a person might believe that effort is not necessary to successfully cope with low demands compared to high abilities, which leads to an increase in performance quantity (as also found in our study), but to a decrease in performance quality (see, e.g., Vancouver et al., 2001, 2002, 2014; Vancouver, 2012). That is why we assumed in *Hypothesis 1b* that we would find a negative indirect effect of positive feedback via self-efficacy on performance quality. However, contrary to *Hypothesis 1b*, we found this indirect effect to be *positive*, with a positive effect of self-efficacy on performance quality. A possible explanation for the contradictory findings in the literature could be that the undermining effect of self-efficacy on performance quality only occurs if self-efficacy is very high. In our case, we told participants in the feedback condition that their performance was “above average”, which is a relatively moderate manipulation. Thus, while we successfully increased self-efficacy levels, they only increased to a moderate but not to a very high level. This explanation would be in line with the proposed inverted u-shaped relationship between self-efficacy and performance (cf. Schönfeld et al., 2017): there could be an increase in performance until self-efficacy is moderately high and a decrease in performance if self-efficacy further increases.

*Hypothesis 2* was also confirmed, with our results suggesting that positive feedback increases flow-experience via increased self-efficacy. The finding that experimentally induced self-efficacy increased flow-experience supports earlier cross-sectional field studies that have found positive associations between the two (Zubair and Kamal, 2015a, b) and longitudinal studies (Salanova et al., 2006; Rodríguez-Sánchez et al., 2011) with first evidence for a causal effect of self-efficacy on flow experience. By using an experimental manipulation to increase self-efficacy, we add further evidence to the existing literature that self-efficacy can causally increase flow-experience.

However, our study did not test the opposite causal direction, that experiencing flow would increase self-efficacy. Flow provides an enjoyable feeling of control over the activity at hand, while applying one’s skills. Thus, it is likely that flow also enhances self-efficacy, and that the effects are bidirectional. This reciprocity suggests that an upward spiral of self-efficacy and flow can occur – as supported by earlier results from field studies (Salanova et al., 2006, 2014). Future experimental studies should look at both causal directions, replicating and complementing previous results.

Our results further showed an indirect effect of positive feedback on flow. This is in line with earlier theory and research: In his nine components of flow-experience, Csikszentmihalyi (1990) had already named “clear goals and feedback” as one of the components. Later operationalizations of flow distinguished between antecedents, characteristics and consequences of flow and considered feedback as an antecedent (Nakamura and Csikszentmihalyi, 2002; Landhäußer and Keller, 2012). Cross-sectional field studies on feedback and flow supported this assumption (Rau and Riedel, 2004; Maeran and Cangiano, 2013). To the best of our knowledge, ours is the first study to show an indirect effect of *positive* feedback on flow in an experimental design, thereby providing insights into a mechanism that can transmit the effects of positive feedback to flow: self-efficacy. Accordingly, providing positive feedback enhances self-efficacy, which presumably enhances the feeling of competency and control in the respective task – two characteristics of flow experience.

However, it needs to be stated that while the indirect effect of positive feedback via self-efficacy on flow was significant, the total effect was not significant, and neither was the direct effect of positive feedback on flow when self-efficacy was included as a mediator. According to Hayes (2009, 2013), finding an indirect effect confirms mediation, while a missing total effect does not contradict mediation: “A failure to test for indirect effects in the absence of a total effect can lead to you miss some potentially interesting, important, or useful mechanisms by which X exerts some kind of effect on Y (Hayes, 2009; p. 415).” In cases in which the total effect is not significant, it is likely that different mechanisms play a role in the relationship between independent and dependent variable. This means that in addition to a positive effect of positive feedback on flow via enhanced self-efficacy, it is likely that there are counteracting mediators in this process, transmitting negative effects of positive feedback on flow. Such possible mediators should be investigated in future studies.

Similarly, the indirect effect of positive feedback via self-efficacy on performance quantity and quality were significant, while the total and direct effects were not significant. This again underlines the possibility of counteracting mechanisms between positive feedback and performance. While positive feedback via self-efficacy positively impacts performance, positive feedback might lead to the assumption that less effort is necessary, which could have a counteracting negative impact on performance (e.g., Vancouver et al., 2014). Such counteracting mechanisms of action should be examined in future studies, for example with the use of physiological indicators of mental effort, such as high frequency heart rate variability. Not only short-term but also long-term perspectives are relevant: an increase in self-efficacy through regular positive feedback might have long-term consequences on flow, and – through extensive application – on skill acquisition and future performance.

Another possible explanation for the lack of a total or direct effect of positive feedback on flow is that the feedback manipulation was potentially not salient enough: We told participants that they were better than average – which is what most people would assume anyway: the “better-than-average” effect is a robust finding in research on social comparisons (compare Alicke and Govorun, 2005). Accordingly, our positive feedback was potentially a confirmation of an existing assumption (i.e., neutral feedback) rather than a positive deviation. Future studies should use stronger positive feedback manipulations in order to investigate its effects on flow and performance.

Yet another explanation for the missing findings could be the kind of feedback that we used: Prior studies that addressed the relationship between flow mostly referred to either task-inherent feedback or supervisor feedback (mostly according to the Job Characteristics Model, Hackman et al., 1975). In their feedback measure, these studies did not differentiate between a normative vs. an individual reference norm (e.g., Bakker, 2005; Demerouti, 2006). Regarding performance there is research differentiating between normative and individual feedback: Brunstein and Hoyer (2002), for example, investigated the effects of positive vs. negative feedback with normative vs. individual reference norm on performance. They did not find any main effects of positive normative feedback on performance (i.e., d2-test for concentration). Descriptively, positive normative feedback was even associated with lower performance (higher reaction times). In addition, Brunstein and Hoyer (2002) found significant interactions with the achievement motive: A higher implicit (but not explicit) achievement motive was associated with higher performance after feedback. Interestingly, it was not positive normative feedback but negative individual feedback that spurred achievement-motivated individuals’ performance. These results show that future studies should explicitly differentiate between normative and individual feedback to get deeper insights into the mechanisms that facilitate or hinder flow experience and performance. As moderating variables, implicit and explicit achievement motives should be controlled, as differential effects might occur. Furthermore, feedback with an individual reference norm may be more appropriate to foster performance and flow (Brunstein and Hoyer, 2002; Brunstein and Maier, 2005).

While Brunstein and Hoyer found that performance was enhanced by negative feedback at least for individuals scoring high on the implicit achievement motive, we do not know how negative feedback impacts flow. In theoretical models (Nakamura and Csikszentmihalyi, 2002; Landhäußer and Keller, 2012) as well as in previous (field) studies (Rau and Riedel, 2004; Maeran and Cangiano, 2013) on the relationship between feedback and flow, positive and negative feedback were not distinguished. However, there is evidence from qualitative research that positive feedback is especially beneficial for flow (Jackson, 1995; Swann et al., 2015). Thus, it will be interesting for future studies to look at the differential effects of positive vs. negative feedback, differentiating between task-inherent, normative and individual feedback, and controlling for an individual’s achievement motive.

A potential limitation of our results refers to the fact that we did not use change scores of performance in the mental arithmetic tasks to control for baseline scores. However, based on the design of our study, the interpretation of change scores is problematic: The two tasks differed very much in difficulty: while the first task was very easy (continuously subtracting 12 from 2000), the second task was more difficult (continuously subtracting 17 from 2043). Our results clearly support this, as both performance indicators decreased significantly from t1 to t2. Furthermore, the low difficulty in the first task likely led to a ceiling effect of performance in both groups, reducing the systematic variance in the data. These circumstances make the change scores very hard to interpret. At the same time, as we had randomly assigned our participants to the experimental conditions, we believe that mental arithmetic skills are equally distributed between groups. Accordingly, we refrained from using change scores in our data analysis. Detailed results using change scores in the analyses can be found in the Supplementary Material.

One more possible limitation of our study refers to the measurement of flow: By using the flow short scale (Engeser and Rheinberg, 2008), we applied a widely used componential approach to assess flow (compare Moneta, 2012). This scale measures flow as a continuous phenomenon: the more its components are pronounced, the higher flow values. The components used in the flow short scale reflect those proposed by Csikszentmihalyi (1975, see flow definition above). However, an ongoing discussion in flow research is whether flow is a continuous phenomenon or if it is rather a yes-or-no phenomenon (compare Engeser, 2012; Peifer and Engeser, in press), with an individual either being in flow or not. A cut-off-value for flow when measured with the componential approach has not yet been identified and is a challenge for future research. With what we know today, the current study is limited insofar as it cannot differentiate between flow or not-flow, but rather measures more or less pronounced flow components.

### Practical Implications

Based on our results, we can recommend positive feedback as an intervention to enhance self-efficacy. This recommendation can be applied to different contexts such as work or schools. In the work context, positive feedback could be given by supervisors in annual performance reviews and in regular meetings. A precondition to providing clear feedback is goal setting, as expectations are made transparent to the employee. Goals should be realistic and achievable in due time in order to provide the opportunity for positive feedback on a regular basis. In general, positive feedback can also come from sources other than just the supervisor – for example from customers or colleagues. Positive feedback has further been found to be positively related to job satisfaction (Yukl et al., 2002) and well-being (Stocker et al., 2014). Thus, an organizational climate of mutual appreciation is a good basis for employees’ self-efficacy, satisfaction and well-being. As shown in the current study, self-efficacy is also positively related to flow and performance and acts as a mediator transmitting positive effects of positive feedback to flow and performance.

While we manipulated self-efficacy in this study using positive feedback, there are other interventions that have been successfully used to enhance self-efficacy, for example interventions to increase psychological capital (Luthans et al., 2006, 2008, 2010). These interventions could therefore also be used to increase self-efficacy, and, in turn, performance and flow-experience.

In this study, we found short-term and immediate effects of self-efficacy on flow-experience. Future studies should have a look at spillover effects and at long-term effects of self-efficacy interventions: Enhancing flow-experience has been found to affect future flow and performance in similar tasks (Christandl et al., 2018). Furthermore, flow has been found to lead to long-term increased performance via increased motivation to practice (Engeser et al., 2005; Schüler, 2007; Schüler and Brunner, 2009). Thus, interventions to foster the pleasant experience of flow are a valuable endeavor for institutions (e.g., organizations, schools) as well as for individuals (e.g., employees, students).

## Conclusion

“Well done?” – our study investigated the relationships between positive normative feedback, self-efficacy, performance, and flow experience. Our results provide experimental evidence that positive feedback enhances self-efficacy. Further, we found an indirect effect of feedback via self-efficacy on performance quantity and quality, as well as on flow experience. However, mutually opposing counter-mechanisms were potentially also active as we did not find a total effect of positive normative feedback on performance and flow, calling for further research on this issue.

## Data Availability Statement

The datasets generated for this study are available on request to the corresponding author.

## Ethics Statement

All procedures performed in studies involving human participants were in accordance with the ethical standards of the institutional and/or national research committee and with the 1964 Helsinki declaration and its later amendments or comparable ethical standards.

## Informed Consent

Informed consent was obtained from all individual participants included in the study.

## Author Contributions

CP and PS conceived of the presented idea. PS carried out the experiment. CP, PS, and FA developed the theory. PS and GW wrote the methods. GW and FA performed the computations and wrote the results part. CP wrote the discussion. JM supervised the concept and findings of this work. All authors discussed the results and contributed to the final manuscript.

## Conflict of Interest

The authors declare that the research was conducted in the absence of any commercial or financial relationships that could be construed as a potential conflict of interest.

## Footnotes

1 We performed all analyses with and without outliers and results did not change.
