# Supplementary material for: Well Done! Effects of Positive Feedback on Perceived Self-Efficacy, Flow and Performance in a Mental Arithmetic Task
Source: Front Psychol. 2020 Jun 10;11:1008. doi: 10.3389/fpsyg.2020.01008 (PMC7300320; doi:10.3389/fpsyg.2020.01008)
Supplement: Supplementary file 1 [file Data_Sheet_1.PDF]

**Supplementary Analyses**

Table: Means and standard deviations of performance quality and quantity at t1 and t2 for the whole sample and separated by groups.

| | Overall | | Experimental group | | Control group | |
|---|---|---|---|---|---|---|
| Variable | MW | SD | $MW_{EG}$ | $SD_{EG}$ | $MW_{CG}$ | $SD_{CG}$ |
| Performance QL t1 | 0.93 | 0.06 | 0.93 | 0.06 | 0.92 | 0.07 |
| Performance QL t2 | 0.91 | 0.10 | 0.91 | 0.10 | 0.90 | 0.09 |
| Performance QN t1 | 40.67 | 10.76 | 41.12 | 10.07 | 40.19 | 11.56 |
| Performance QN t2 | 28.53 | 9.19 | 28.35 | 8.68 | 28.73 | 9.80 |

*N* = 100 (EG=52; CG=48); *t1* = after the first task; *t2*= after the second task

**Testing the differences in performance at t2 compared to t1**

To test for performance differences at t2 compared to t1, we performed two repeated measures ANOVAs. After exclusion of two outliers on the performance variables at t1, the sample size for these ANOVAs was 100. For performance quantity, the effect of time was significant ($F_{(1, 98)} = 310.97, p < .001, \eta_p^2 = .760$), showing that performance quantity decreased from t1 to t2. The interaction between experimental and control group was not significant ($F_{(1, 98)} = 0.91, p = .342, \eta_p^2 = .009$). For performance quality, effects were similar, with a significant effect of time ($F_{(1, 98)} = 4.82, p = .030, \eta_p^2 = .047$), showing that performance quality decreased from t1 to t2. The interaction between experimental and control group was not significant ($F_{(1, 98)} = 0.27, p = .608, \eta_p^2 = .003$). While both performance quantity and quality were decreased in t2, this results can very likely be explained by the higher degree of difficulty of the second task. While the first task was very easy (continuously subtracting 12 from 2000), the second task was more difficult (continuously subtracting 17 from 2043). Thus, it is very likely that there are ceiling effects regarding performance in the first task in both groups, reducing the systematic variance in the data and making the change scores hard to interpret. Accordingly, we refrain from using change scores in our data analysis.

**Testing Hypotheses using change scores for performance and self-efficacy**

To test *Hypothesis 1a*, we z-standardized all study variables and performed a mediation procedure with feedback as the independent variable, the change in specific self-efficacy (t1 – t0) as the mediator, and the change in performance quantity (t2 – t1) as the dependent variable. The *a*-path ($\beta$ =0.75, $SE$=0.19, $p$<.001) was significant but the *b*-path ($\beta$ =0.00, $SE$=0.11, $p$ = .984) was not significant. The indirect effect was $\beta$ =0.00 ($SE$=0.08, $-0.16 < CI < 0.17$) and not significant. The total effect was $\beta$ =-0.19 ($SE$=0.20, $t$=-0.95, $p$=.342) and not significant. The direct effect was also not significant ($\beta$ =-0.19, $SE$=0.22, $t$=-0.89, $p$=.378) when controlling for the indirect effect. Accordingly, the feedback affected the change in self-efficacy (a-path), but the change in self-efficacy did not affect the change in performance quantity (b-path). As described above, this might be a result of the large differences in task difficulty between t1 and t2.

For Hypothesis 1b we found similar results. To test *Hypothesis 1b*, we again z-standardized all study variables and performed a mediation procedure with feedback as the independent variable, the change in specific self-efficacy (t1 – t0) as the mediator, and the change in performance quality (t2 – t1) as the dependent variable. The *a*-path ($\beta$ =0.75, $SE$=0.19, $p$<.001) was significant but the *b*-path ($\beta$ =-0.11, $SE$=0.11, $p$ = .308) was not significant. The indirect effect was $\beta$ =-0.08 ($SE$=0.09, $-0.28 < CI < 0.09$) and not significant. The total effect was $\beta$ =-0.10 ($SE$=0.20, $t$=-0.51, $p$=.608) and not significant. The direct effect was also not significant ($\beta$ =-0.02, $SE$=0.22, $t$=-0.09, $p$=.929) when controlling for the indirect effect. Again, the feedback affected the change in self-efficacy (a-path), but the change in self-efficacy did not affect the change in performance quality (b-path). Again, and as described above, this might be a result of the large differences in task difficulty between t1 and t2.
